# Rotary 3D Magnetic Field Scanner for the Research and Minimization of the Magnetic Field of UUV

**DOI:** 10.3390/s23010345

**Published:** 2022-12-29

**Authors:** Karol Jakub Listewnik, Kacper Aftewicz

**Affiliations:** Department of Ship Electrical Power Engineering, Faculty of Marine Electrical Engineering, Gdynia Maritime University, Morska St. 83, 81-225 Gdynia, Poland

**Keywords:** magnetometer, 3D magnetic field, magnetic field scanner

## Abstract

Research on the value and nature of physical quantities allows for a detailed understanding of the conditions in the studied area, and the quality and precision of the final conclusions depend on the accuracy of the measurements. In order to increase the accuracy of measurements, the measurement infrastructure and unmanned vehicles used during the observation should introduce the lowest possible disturbance–they should be minimized in terms of the magnetic field. This article presents a solution based on the infrastructure model and the development of a method using polynomial regression to study the magnetic field in three dimensions (3D-longitudinal *X*, transverse *Y*, and vertical *Z* components). The test stand consists of an Arduino Mega microcontroller, a rotary table driven and controlled by a stepper motor, a touch display whose task is to control the magnetic field measurement parameters and display 3D data, and proprietary software made in the Python programming language. The structural elements of the stand model were produced by an additive method using a 3D printer. The presented solution belongs to the group of modern technological solutions known as the technology of low object detection (stealth technology or low observable technology).

## 1. Introduction

The most of the world’s navies have the infrastructure for precise three-axis magnetic field measurement of ships and marine equipment, ROVs and UUVs, and surface USVs [1]. This measurement is usually carried out by two methods, related to the fact that the magnetic, electric, hydroacoustic, and hydrodynamic fields (signatures) are measured simultaneously:

- Measurement using special infrastructure on land: only the magnetic field (and partial airborne equipment acoustic testing [2]), as the other fields require the presence of a water medium;
- Measurement in the sea on special measurement ranges, where the ship’s route is marked with buoys or guides and supported by GPS systems (measuring equipment is placed on the bottom of the sea basin or mounted on AUVs [3,4]).

**Figure 1:**
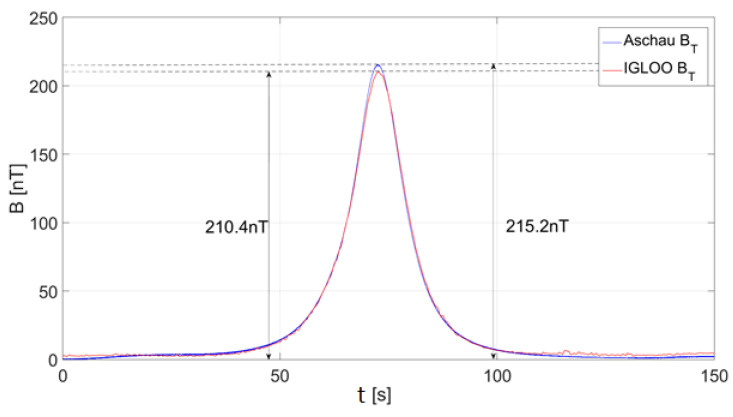
Vector diagram of the total magnetic field (B) of a small steel ship, length 20 m, speed 2.6 m/s, course ~230°. The three-axis magnetic field sensors were used for the measurement by two independent measurement systems: Aschau (WTD71 reference system, German) and IGLOO (Polish). Relative measurement error of the maximum value is 2.2% (author’s source).

An example of a comparative measurement of the magnetic field at the WTD71 test site in Aschau, Germany, is shown in Figure 1. The infrastructure described above is very expensive, and research conducted using it both on land and at sea is expensive and time-consuming. These tests are usually carried out in order to ensure low detectability of ships and underwater vehicles and protection against the mine threat, and this is called passive defense of the ship. Usually, test reports are classified. Currently, the use of submersible vehicles goes far beyond military applications to many civilian applications, which will be briefly discussed below. Current electronic and IT technologies enable access to the development of tools for much cheaper and less time-consuming measurement of the magnetic field of underwater vehicles, their parts, and equipment.

The development of robotic technologies over the last decades has led to a broadening of the scope of research and environmental observations [5]. Numerous applications of UXV unmanned systems can provide valuable data, for example, by UUV in a hard-to-study marine environment [6] due to, for example, turbidity in the water or objects buried in the bottom or hidden in silt. Important areas are clearance of contaminated sites in the sea like clearance of unexploded ordnance and chemical munitions [7] and electromagnetic fields (EMF), which determine the exposure of marine organisms to magnetic and induced electric fields from undersea power cables: (1) the amount of electrical current being carried by the cable, (2) the design of the cable, and (3) the distance of marine organisms from the cable [8] and use of magnetometers in underwater archaeology [9].

Various types of sensors are used in these tests, such as magnetic field sensors (3D or one axis, north indicator), cameras, thermometers, as well as hydro acoustics (e.g., echosounders, sonars, hydrophones), pressure, or electric field sensors. All these physical quantities improve our understanding of the conditions prevalent in an area under study, while the quality and precision of the final conclusions depend on the accuracy of the measurements. To increase measurement accuracy, the measurement infrastructure and unmanned vehicles used during the observation should cause the least possible disturbance [10].

The focus of this work was the magnetic field, and a model of a test stand for measuring magnetic properties of materials is suggested, which may be used to build unmanned vehicles for research and environmental observation in the future. The purpose of this stand is to gain experience and test the applied solutions that can be used in the future during the construction of a full-scale UUV test stand.

The magnetic field is a state of space where forces act on objects with a non-zero magnetic moment, such as permanent magnets, and on moving electric charges such as electrons. Our everyday natural source of magnetic field is the Earth [11]. In addition, magnetic minerals such as magnetite and metal ores such as iron also produce a natural magnetic field. By placing a ferromagnetic material in a magnetic field, the magnetic domains in the material align with the direction of the external magnetic field, thereby amplifying it. After the disappearance of the external magnetic field, the magnetization of the material remains, maintaining the field [12]. Disturbances in the natural magnetic field are used to detect invisible objects with ferromagnetic properties, e.g., buried mines.

## 2. Description of the Test Stand

**Figure 2:**
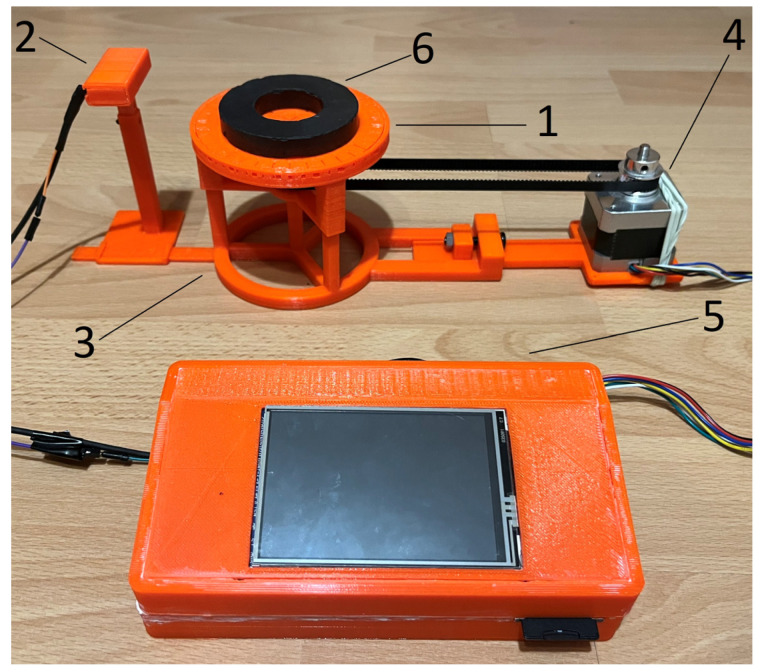
The measuring stand with individual elements distinguished: the rotating stand (1), the magnetometer on an adjustable holder (2), the ruler marking the distance from the center of the rotating base (3), the stepper motor with a drive belt (4), the control module with a display (5), and the tested toroidal magnet (6).

The stand used to carry out the measurements is a model of a stand that is scalable, and the experience gained will be used to build a full-scale measuring stand for studying the magnetic induction distribution of unmanned vehicles, their components, and electrical devices. The stand (Figure 2) consists of (1) a rotating base on which the tested object is placed, (2) a sensor located on an adjustable handle (at an adjustable height and distance from the center of the rotating base, (3) a ruler indicating the measurement distance to the center of the rotating base, (4) a stepper motor with a belt driving the rotary stand, and (5) a central module with a display that controls the operation of the device. The solution is based on earlier solutions for monitoring selected physical quantities in real time [13,14].

**Figure 3:**
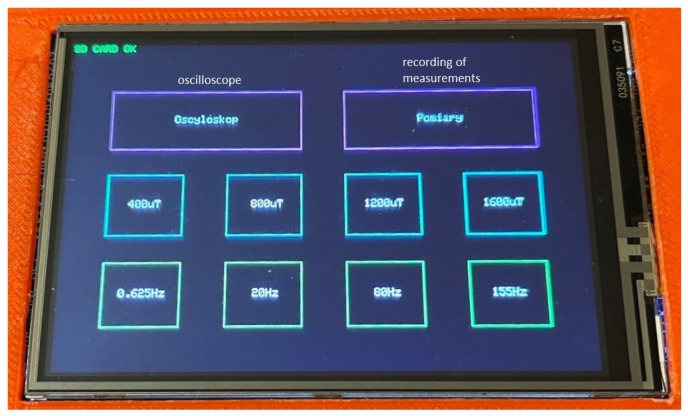
Screenshot with the option selection menu.

A three-axis magnetic induction sensor LIS3MDL [15], made in MEMS technology using the Hall effect, was used as the magnetic field sensor (the magnetic sensor can be selected depending on the needs). It has configurable scopes of measurement in the range of ±400 μT, ±800 μT, ±1200 μT, and ±1600 μT. The magnetometer has a built-in 16-bit analog-to-digital converter (ADC). The highest measurement accuracy possible is in the measuring range of ±400 μT; in this range, the measurement resolution is 0.012 μT [16]. The device has two operating modes: automatic and graphic (Figure 3). By using a rotating base, in automatic mode, measurements are made in the range of 360° in 9° increments, while the device is operated and data are recorded without the involvement of an operator. Measurements are made five times in the full base rotation range, which results in five full rotations of the tested object in relation to the magnetometer. Each measurement is saved on an SD memory card.

**Figure 4:**
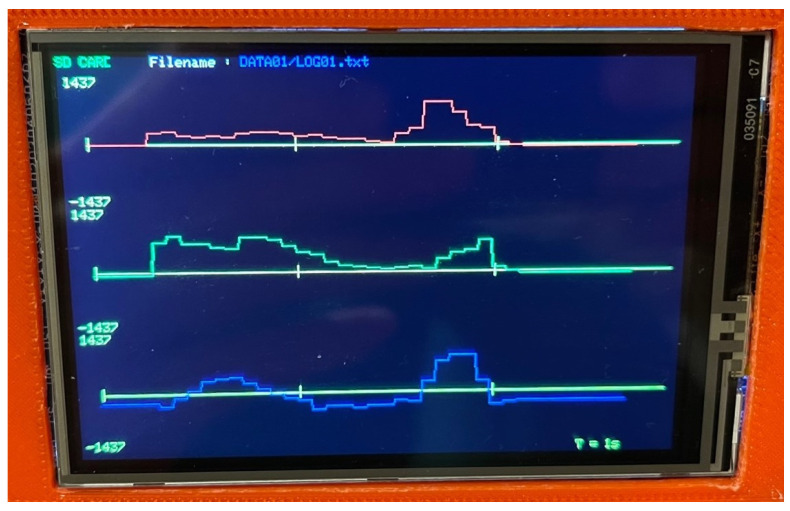
Sample diagram of the magnetic field.

The graphic mode enables the magnetic induction values to be displayed in real time (Figure 4) in three measurement axes and the data to be saved on a memory card. The rotating stand is then inactive, and the operator himself decides when to start and stop recording the measured values. Before the device starts running, a configuration window appears where the user selects the measuring ranges, operating mode and sampling frequency. The option to select the sampling frequency is available only when working in graphic mode, since it presents the current waveforms of changes in the field value in real time. The possible sampling frequencies are 0.625 Hz, 20 Hz, 80 Hz, and 155 Hz.

The sensor and other electronic components are integrated with the Arduino Mega platform, and the entire measuring system is controlled by a proprietary program written in the Arduino language.

Most of the stand’s elements were made via PLA 3D printing because it is a non-magnetic material, thereby restricting measurement errors from the measuring stand. The stepper motor (Figure 2 (4)) is extended so far that it does not affect the measured values.

## 3. Research Methods

The method used to determine the distribution of magnetic induction of magnetic objects, which is based on measurements at several selected points at any distance from the measuring range and on calculations using polynomial regression, is presented below using ferrite permanent magnets, commonly used as a compromise between financial and technical considerations. Selected values of the parameters characterizing ferrite magnets are presented in Table 1:

Measurements were made in the laboratory at room temperature (~21 °C), and the magnets were placed directly on the rotating base (Figure 2 (1)). The relationship between the magnetic induction *B* and the magnetic field strength *H* depends on the medium in which the magnetic field is present, as described by Equation (1). The magnetic permeability of a vacuum is a physical constant *µ*_0_, while the relative magnetic permeability of the medium µr characterizes the medium in which the magnetic field is present. For water and air it is assumed that *µ_r_* = 1.0 [11].

(1) $$B={µ}_{r}{µ}_{0}H$$

**Figure 5:**
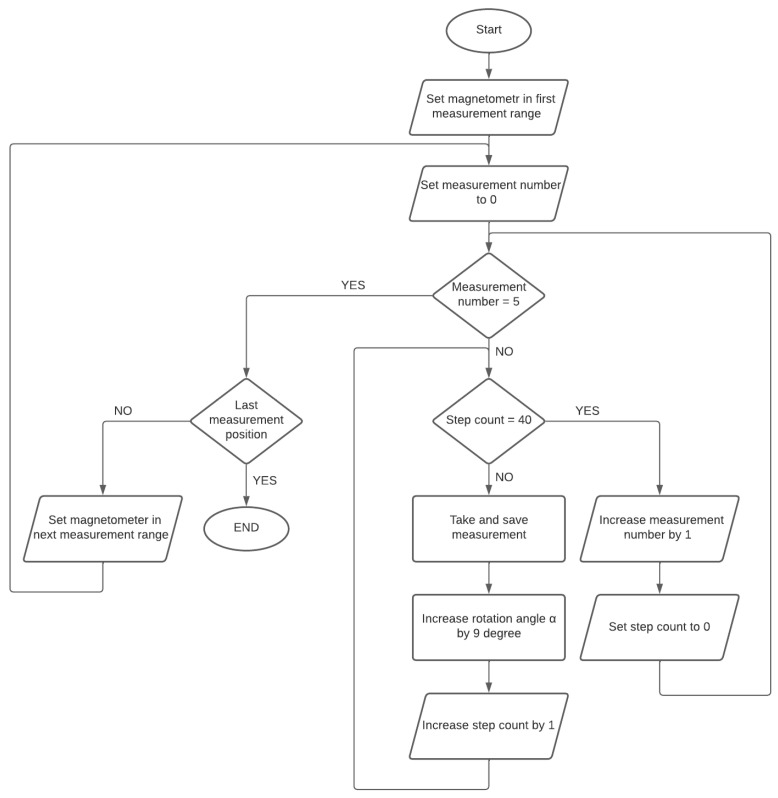
The measurement algorithm for the tested object.

The measuring stand has an automatic mode for taking measurements. Before starting the operation of the device, the tested system of magnets was placed on a rotating stand, described in more detail later in this work. The system was placed in such a way that the rotation of the base was as symmetrical as possible and there were no significant changes in the distance between the system and the magnetometer caused by the rotation of the base. In the automatic operating mode, the base rotates 360° 5 times in 9° steps, and each measurement is saved on the SD card. After making five series of measurements for one position of the magnetometer, the sensor was moved to the next measurement position, and the process of five series of measurements was performed again. The distances at which the measurements were made were, respectively, 6 cm, 7 cm, 8 cm, 9 cm, 10 cm, 11 cm, 12 cm, and 15 cm from the center of the rotating base. Measuring ranges at each distance were selected so as to obtain the highest measurement accuracy without exceeding the allowable measuring range. With the increase in the distance, the measuring range decreased, because the measured magnetic induction reached a lower value. The same steps should be repeated for the magnets or devices selected for testing. The measurement scheme is shown in Figure 5.

## 4. Calculation Method

Non-linear regression is a method that approximates an unknown function f(x) using an estimator. The estimator can be any polynomial function *p*(*x*), *n*–of degree (Equation (2)). It boils down to determining the coefficients of the polynomial in such a way as to reproduce the course of the function f(x) as precisely as possible, i.e., to minimize the error *R*. The least squares method (Equation (4)) is most often used as a measure of the error *R*. Given the values of the function f(x) for the known arguments $x_{i}$ and the calculated values of the estimator values at these points, the difference between the squares of both values is determined, and then the sum of the differences from each known point is made.

(2) $$p\left(x\right)=a_{n}x^{n}+a_{n-1}x^{n-1}+\ldots +a_{1}x+a_{0}$$

The above formula can be represented as a sum and takes the form:

(3) $$p\left(x\right)=\overset{n}{\underset{i=0}{\sum }}a_{i}x^{i}$$

The method of least squares used to determine the error takes the form:

(4) $$R=\overset{n}{\underset{i=0}{\sum }}{\left[\varphi \left(x_{i}\right)-f\left(x_{i}\right)\right]}^{2}$$

where:

φ(x) is the approximating function; and f(x) is the approximated function.

**Figure 6:**
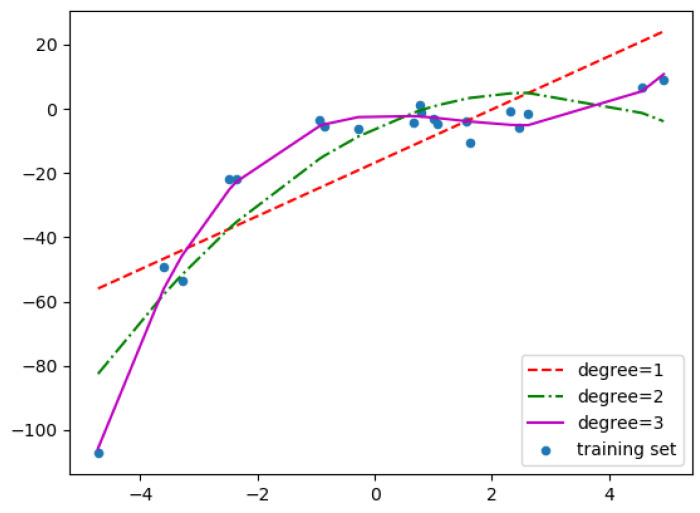
Comparison of the accuracy of the approximation of the function f(x) depending on the degree of the approximating polynomial.

Depending on the degree of the polynomial, the regression can be linear or non-linear. Linear regression uses a zero or first order polynomial, and the effect is to determine the approximating function φ(x) in the form of a linear function. In order to approximate more complex functions, higher degree polynomials are used, which allow for more accurate determination of the value of the function f(x) and minimization of the error *R* (Figure 6) [19].

**Figure 7:**
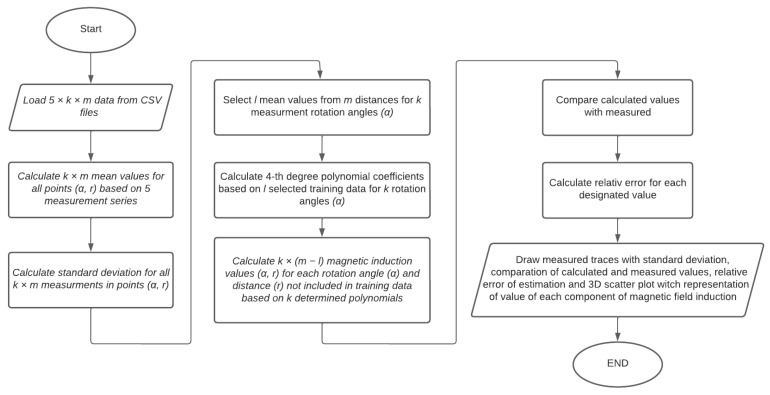
Scheme of calculations and data flow during magnetic induction measurements.

The polynomial regression method was chosen to estimate the distribution of the magnetic field induction due to the heterogeneous nature of the field generated by the tested objects. For each angle of rotation α of the rotating base, an approximation polynomial is determined on the basis of measurements made at m measuring distances for a given angle α. Then, using the determined polynomials, the value of magnetic induction at any distance from the center of the rotating base is calculated. In this way, the induction distribution in the range of 360° is determined in steps of 9°. For calculations and visualization of measurements, a Python script was written using the described method of polynomial regression to determine the magnetic induction of the field at a distance r from the center of the tested object. The data flow is shown in Figure 7.

The first stage of the algorithm is to calculate the resultant value of magnetic induction based on the measured values in three measurement axes in accordance with Equation (5) [11].

(5) $$B=\sqrt{B_{x}{}^{2}+B_{y}{}^{2}+B_{z}{}^{2}}$$

where:

$B_{x}$ is the value of the *X*-axis component of the sensor;
$B_{y}$ is the value of the *Y*-axis component of the sensor; and
$B_{z}$ is the value of the *Z*-axis component of the sensor.

Then, average values are determined for the measurements made for *k* values of the angle α in the range of 0°–360°, equal to the number of positions of the rotational base in which the measurements were made. As the measurements in the automatic mode are performed five times, the calculation of the average value boils down to the sum of the values of five measurements made for each angle α, dividing the values by the number of measurements. The obtained value is the average value of the magnetic induction at the point described by two values: the angle of rotation of the base α and the measurement distance from the center of the base *r_p_*. The averaging process is performed for all measured magnetic induction values, and the standard deviation of the measurements is calculated. The data obtained in this way form a table in which the number of rows corresponds to the number of measurement distances *m*, while the number of columns is equal to *k*, and the number of positions of the rotary base for which the measurements were made. In the next stage of the algorithm, the data prepared in this way are used to determine the coefficients $a_{n}$ and the approximating polynomial of the *n*^th^ degree. For this purpose, *l* measurement values are selected from m available values from each column of the table, in which measurements for one angle α are stored. On the basis of these values, a polynomial approximating the magnetic induction distribution as a function of the distance from the base center is determined for each value of the rotation angle α. For this purpose, the *polyfit* method available in the *numpy* library [20] was used, which implements the nth degree polynomial approximation method using the least squares error minimization described earlier.

On the basis of the obtained approximating polynomials, the values of the magnetic induction of the tested object at distances of 6 cm–15 cm from the center of the base were determined, which are presented in the next section in the form of 3D diagrams prepared using the library *matplotlib.*

### 4.1. Study of the Influence of the Degree of the Approximating Polynomial on the Quality of the Obtained Results

**Figure 8:**
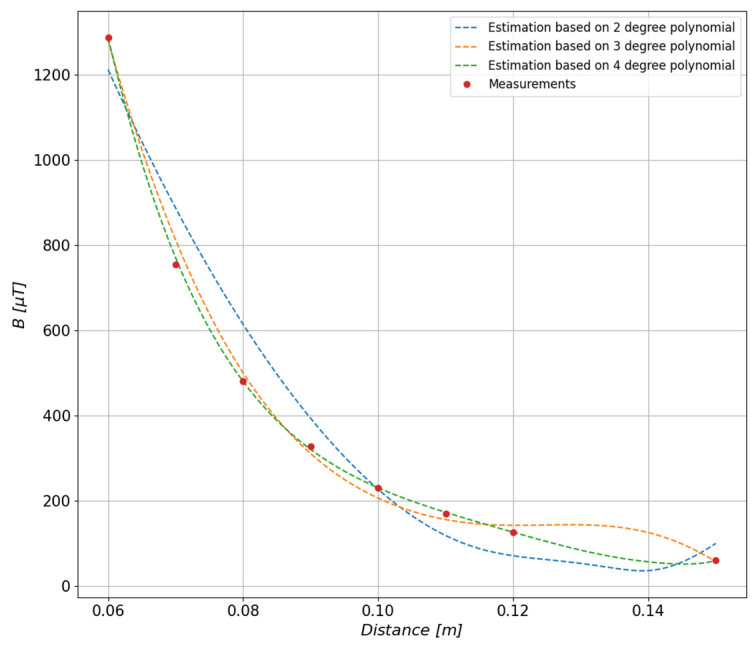
Adjustment of the estimated values depending on the degree of the polynomial determined on the basis of the values measured at the distances of 6 cm, 8 cm, 10 cm, 12 cm, and 15 cm.

When approximating the distribution based on the measured values of the magnetic field, the degree of the approximating polynomial has a large impact on the quality of mapping the value of the field generated by the tested object. The measurements referred to in this article were carried out at eight measurement distances, of which three values were used as controls, which means that they were not used during the determination of the polynomial. In order to select the degree of the polynomial, an iterative method was used consisting of determining several polynomials of the second, third, and fourth degree. Based on the obtained results, the fourth-degree polynomial was selected because it most accurately reproduced the course of values at the control points, which is shown in Figure 8.

**Figure 9:**
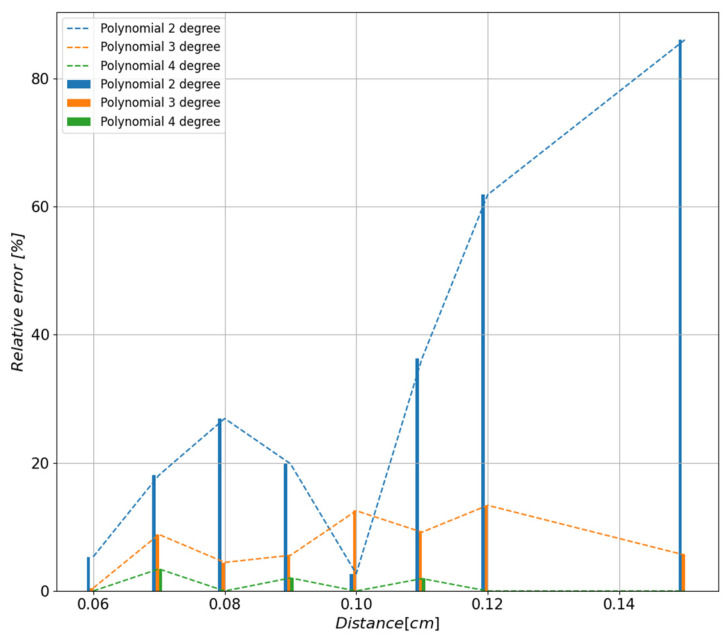
Values of the relative errors depending on the degree of the polynomial determined on the basis of values measured at distances of 6 cm, 8 cm, 10 cm, 12 cm, and 15 cm.

In order to determine the accuracy of the approximation depending on the degree of the polynomial, the relative errors (Figure 9) of the fit of the function to the measurement points presented in Figure 8 were calculated. On this basis, the fourth degree of the polynomial was selected as the function that most accurately represented the measurement points; the relative error does not exceed 3.2%.

### 4.2. Study of the Impact of Data Selection on the Quality of the Approximating Polynomial

The measurements carried out provided a large number of measured values, which then need to be processed. The data recorded by the sensor consists of a three-dimensional array of 5 × *k* × *m*, where *k* is the number of all positions of the angle of rotation α and m is the number of measurement distances at which the measurements were made. According to the previously discussed scheme, the first stage of calculations focuses on determining the average values at each point based on five measurements. After averaging these values, a two-dimensional *k* × *m* matrix is obtained. In order to demonstrate the impact of the selection of measurement points on the quality of estimation of the measured magnetic induction distribution, data for the rotation angle of 27° were selected from the calculated average values, presented in Table 2.

**Figure 10:**
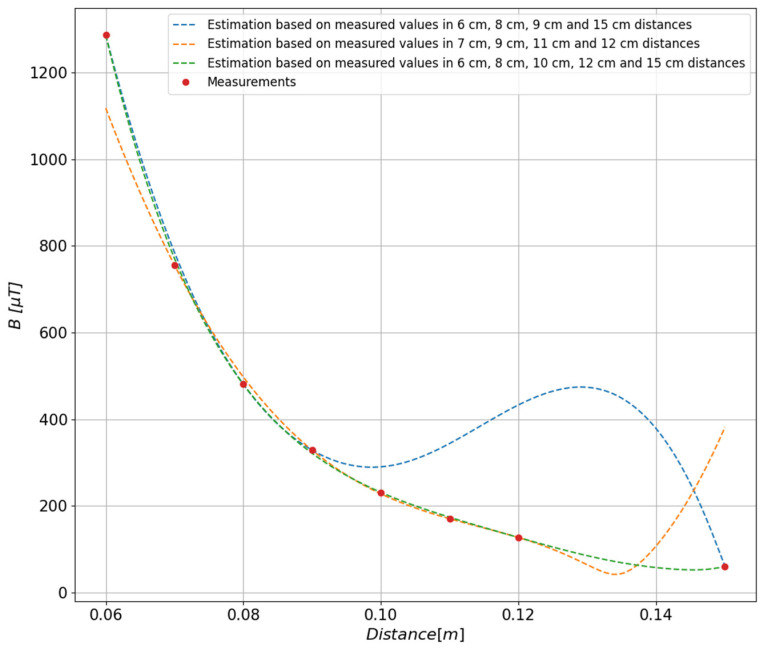
Comparison of three approximating polynomials determined on the basis of different training vectors.

The impact of data selection on the quality of the approximating polynomial is shown in Figure 10. Three approximated waveforms based on different learning vectors have been presented, it can be seen that the green approximating waveform covering five points from the entire range of measurement distances most accurately reflects the waveform obtained from the measurement values.

## 5. Results

### 5.1. Study of the Toroidal Magnet

In order to verify the rotary 3D magnetic field scanner and estimate the obtained accuracy of the determination of the 3D magnetic induction, measurements were carried out with the use of a toroidal permanent magnet. Measurements were performed in the range of 360° every 9°, and the obtained results were saved on a memory card and calculated using the previously described method. 

The ferrite toroidal magnet was placed on the test stand as shown in Figure 2 (6). Magnetic induction measurements were made at a distance of 6 cm, 7 cm, 8 cm, 9 cm, 10 cm, 11 cm, 12 cm, and 15 cm from the center of the rotating base.

**Figure 11:**
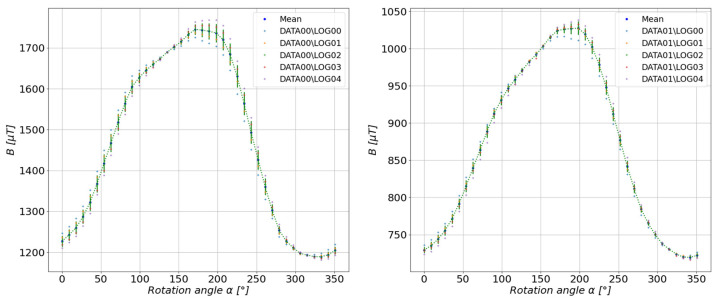
Measurement results at a distance of 6 cm (**left**) and 7 cm (**right**) from the center of the tested configuration.

**Figure 12:**
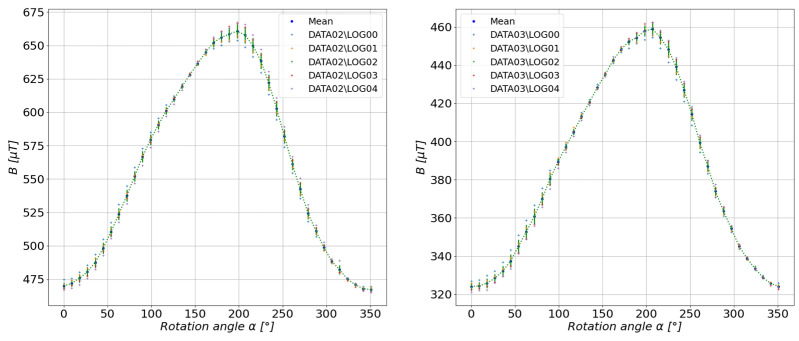
Measurement results at a distance of 8 cm (**left**) and 9 cm (**right**) from the center of the tested configuration.

**Figure 13:**
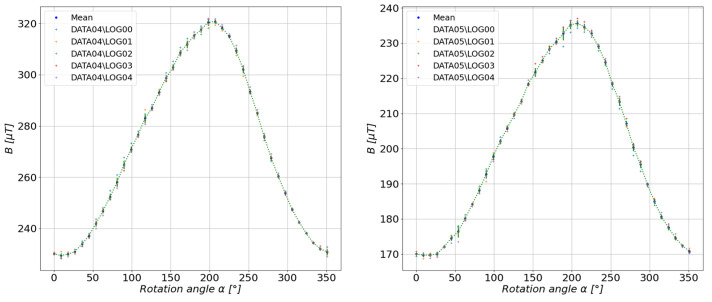
Measurement results at a distance of 10 cm (**left**) and 11 cm (**right**) from the center of the tested configuration.

**Figure 14:**
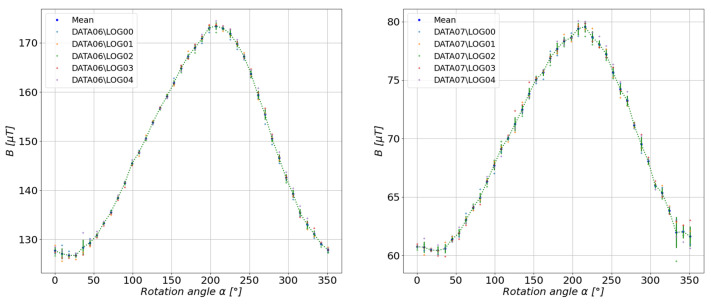
Measurement results at a distance of 12 cm (**left**) and 15 cm (**right**) from the center of the tested configuration.

**Figure 15:**
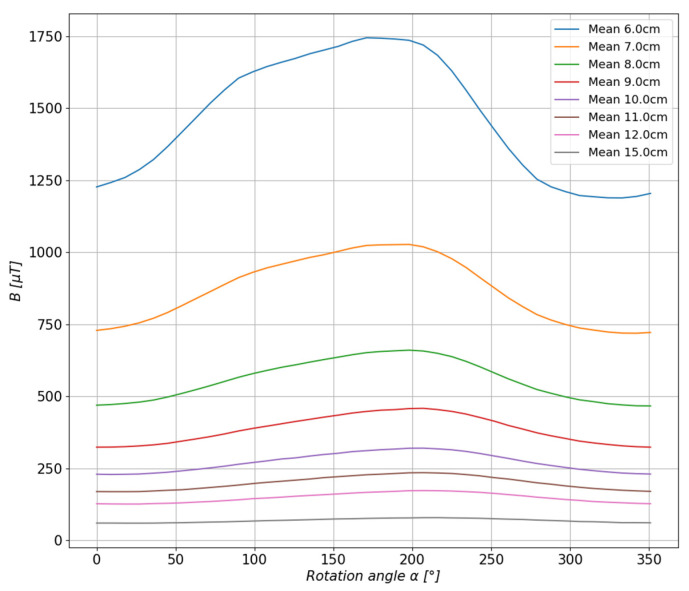
Summary of the measured mean values of the total magnetic induction *B* of a ferrite toroidal magnet.

According to the diagram discussed in the previous section, the mean scalar values were determined for each series of measurements performed at select measuring distances. The results are shown in Figure 11, Figure 12, Figure 13, Figure 14 and Figure 15.

**Figure 16:**
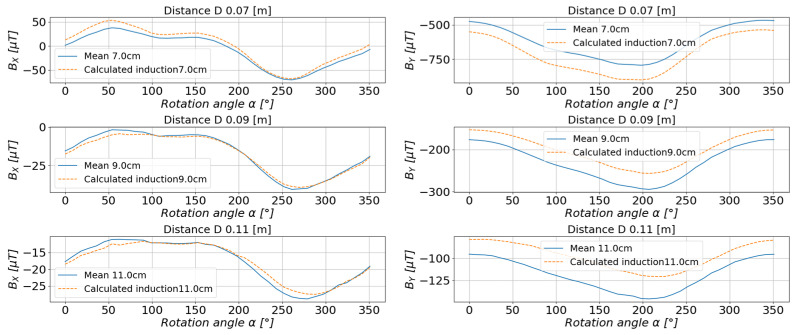
Comparison of the estimated values with the measured values of magnetic induction in the measurement axis *X* (**left**) and axis *Y* (**right**).

**Figure 17:**
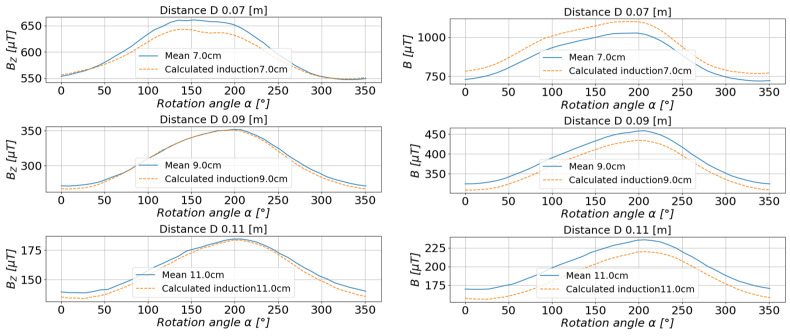
Comparison of the estimated values with the measured values of the magnetic induction in the measurement axis *Z* (**left**) and the resultant value of the scalar magnetic induction *B* (**right**).

For the rest of the calculations, the measurements taken at a distance of 6 cm, 8 cm, 10 cm, 12 cm, and 15 cm from the center of the base were selected. The values of the magnetic induction were calculated, and the comparison of the calculated values with the measured values is presented in Figure 16 and Figure 17.

**Figure 18:**
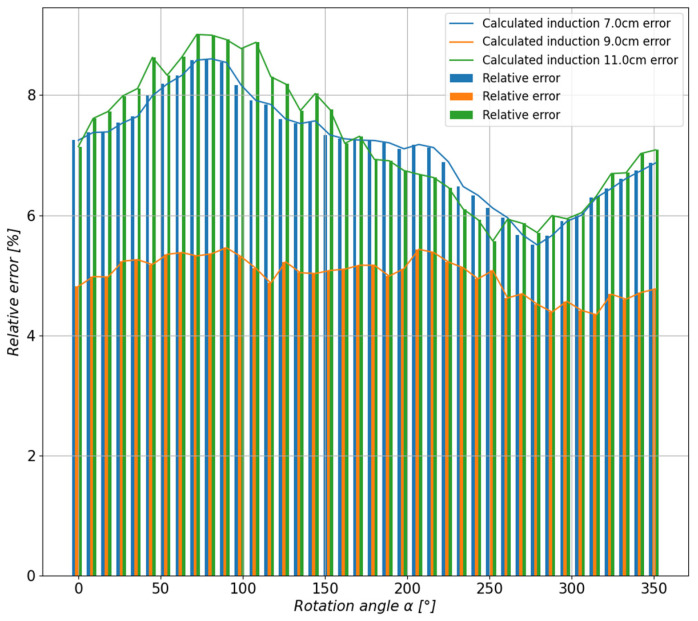
Comparison of the relative errors for the estimated values of the magnetic induction of the tested toroidal magnet depending on the measuring distance.

In order to assess the accuracy of the method and the representation of the actual measured values, the relative error of each of the estimated waveforms as a function of the stand rotation angle was calculated (Figure 18).

**Figure 19:**
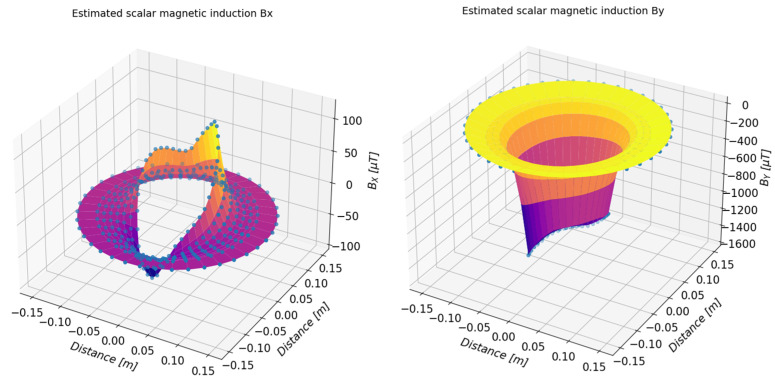
Three-dimensional presentation of the estimated value of the *B_x_* (**left**) and *B_y_* (**right**) components of the magnetic induction of the examined magnets determined on the basis of the measurements.

**Figure 20:**
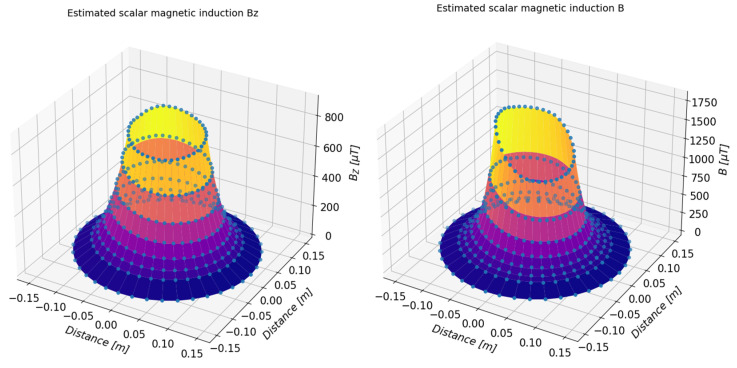
Three-dimensional presentation of the estimated value of the component *B_z_* (**left**) and total value *B* (**right**) of the magnetic induction of the examined magnets determined on the basis of the measurements.

On the basis of the determined approximating functions, the distribution of the magnetic induction of the tested object can also be presented in a three-dimensional form. Figure 19 and Figure 20 present a summary of the measured values (marked with dots) and a three-dimensional distribution of magnetic induction components in the form of a three-dimensional surface. Table 3, Table 4, Table 5 and Table 6 present numerical data from the measurements of the magnetic field of the toroidal magnet.

On the basis of the conducted research, it can be concluded that the detection range of ferromagnetic objects, which are most often used in the marine environment, is comparable to the size of this object.

### 5.2. Study of a Group of Magnets Arranged in Irregular Form

**Figure 21:**
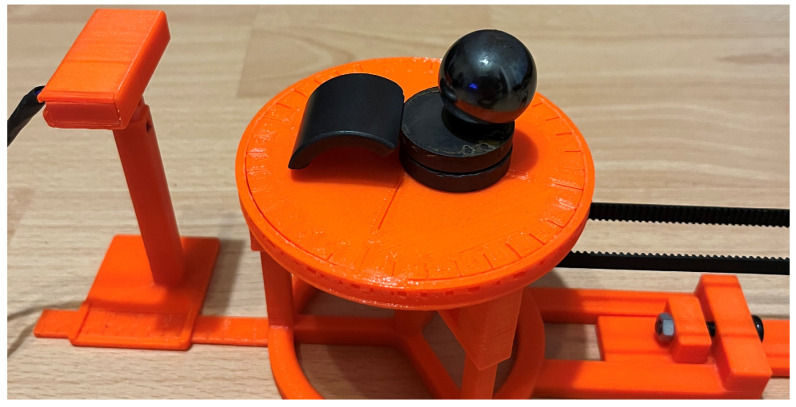
A system of several permanent magnets used during measurements of an irregular magnetic field.

According to the methods of magnetic field measurement and polynomial approximation described above, the distribution of the magnetic field of the system of several permanent magnets presented in Figure 21 was determined.

**Figure 22:**
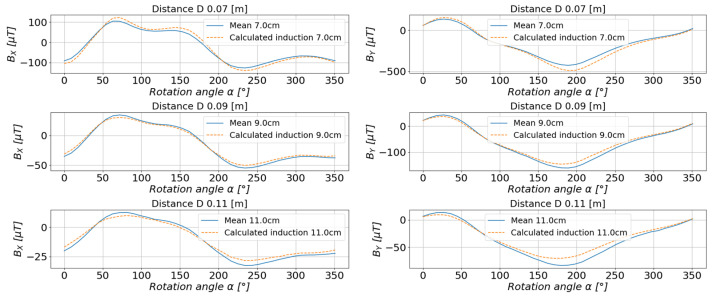
The comparison of the estimated values with the measured values of the magnetic induction in the measurement axis *X* (**left**) and axis *Y* (**right**).

**Figure 23:**
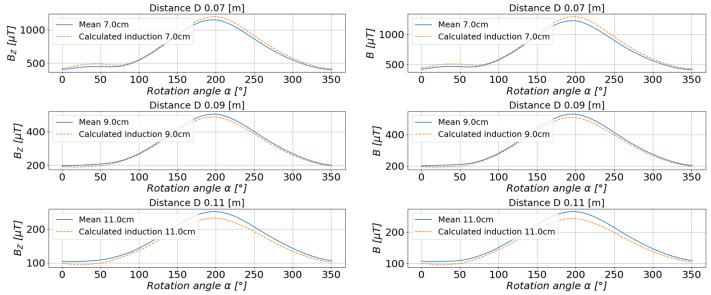
Comparison of the estimated values with the measured values of the magnetic induction in the measurement axis *Z* (**left**) and the resultant value of the scalar magnetic induction *B* (**right**).

The comparison of the obtained results of the estimated and measured values for the system of magnets shown in Figure 21 is shown in Figure 22 and Figure 23.

**Figure 24:**
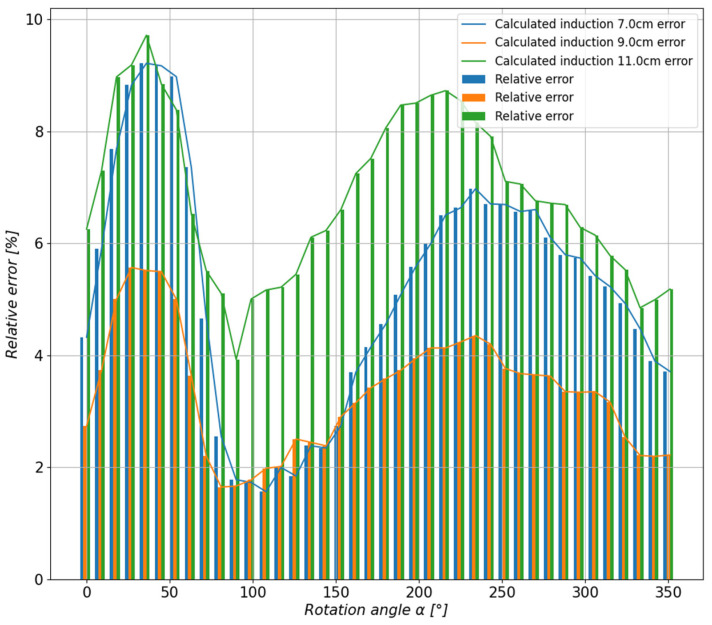
The comparison of the relative errors of the estimated values at the control points of the permanent magnet system.

In order to assess the quality of the approximation, the value of the relative error was calculated, shown in Figure 24 in the form of a bar graph, for each control distance as a function of the base rotation angle.

**Figure 25:**
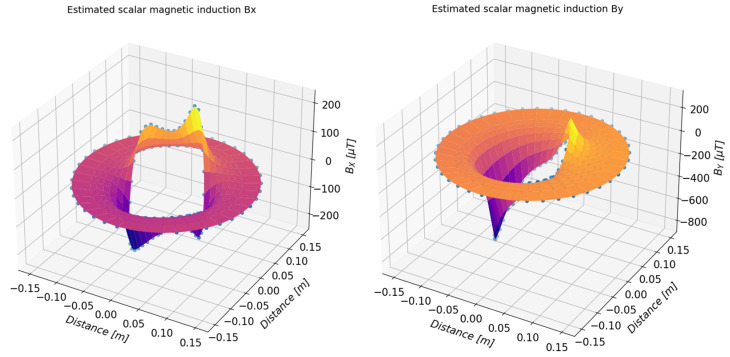
The three-dimensional view of the estimated value of the *B_x_* (**left**) and *B_y_* (**right**) components of the magnetic induction of the examined magnets determined on the basis of the measurements.

**Figure 26:**
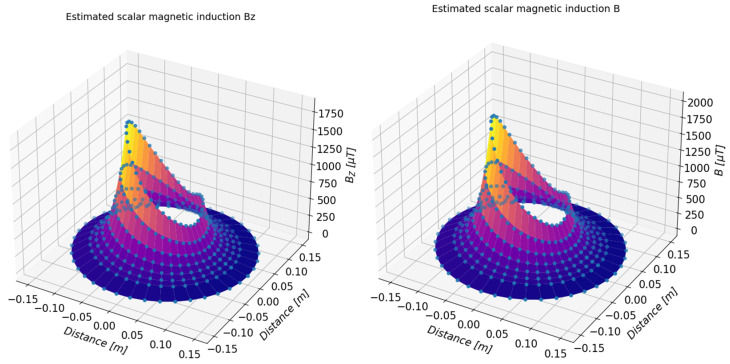
The three-dimensional view of the estimated value of the component *B_z_* (**left**) and total value *B* (**right**) of the magnetic induction of the examined magnets determined on the basis of the measurements.

The obtained measurement results are presented in the form of 3D graphs in Figure 25 and Figure 26 and in tabular form in Table 7, Table 8, Table 9 and Table 10.

Based on the data in Table 6 (regular toroidal shape of the magnet) and Table 10 (irregular shape of the set of magnets), it can be seen that for the doubled measuring distance (between 6 cm and 12 cm) there is a tenfold decrease in the value of the total magnetic induction *B*, which means that at this distance, an object with magnetic properties is much more difficult to detect.

## 6. Conclusions

At present, measurements of the physical fields (magnetic, pressure, seismic, acoustic, and electric) of UUV underwater unmanned vehicles are not commonly used, primarily due to the lack of availability of appropriate research positions and qualified research staff. Such tests are carried out sporadically, which significantly affects the quality of the components of these vehicles and the general characteristics (so-called objects or ship signatures) of the UUV physical fields.

The stand prepared in this work and the conducted research indicate that the proposed solution meets the assumed requirements. The accuracy obtained, estimated on the basis of the relative error between the values obtained from the measurements and the values calculated using polynomial regression, is sufficient and reaches 9% in the worst case. The test stand is scalable, i.e., using a different (larger) design and more advanced three-axis magnetic field sensors, the proposed method and software can be used without any changes. An important issue is to popularize the proposed solution. It has to be taken into account that depending on the application, the minimization criteria may be different. For example, UUV minimization for the purpose of detecting disorderly intruders and protecting the so-called critical infrastructure will require such optimization of the vehicle’s structural elements so that its magnetic field is within or slightly greater than the values of the three components of the Earth’s magnetic induction at the site of the mission. On the other hand, the UXV task consisting of the detection of magnetic materials in a given environment (e.g., for the neutralization of ammunition or archaeological research) will require detailed knowledge of the distribution of the UUV magnetic field and possible software corrections related to the magnetic induction value of the detected objects with magnetic properties. 

## Figures and Tables

**Table 1:** Selected parameters of ferrite magnets [17,18].

Symbol of the Material	Remanence *B_R_* [mT]	Coercive Field *H_C_* [kA/m]	Energy Density *B_Hmax_* [kj/m^3^]	Curie Temperature *T_C_* [°C]
F10 (isotropic)	200–235	210–280	6.5–9.5	450
F20	320–380	140–195	18.0–22.0	450
F25	360–400	140–200	22.5–28.0	450
F30	370–400	180–220	26.0–30.0	450
F30BH	380–400	235–290	27.0–32.5	450
F35	410–430	225–255	31.5–35.0	450

Note: Remanence *B_R_*: the value of the magnetic induction remaining after the removal of the external magnetic field [T]; Coercive field *H_C_*: the value of the external magnetic field that reduces the remanence to zero [A/m]; Energy density *B_Hmax_*: momentary value of the magnetostatic energy accumulated in the magnetic material [kJ/m^3^]; Curie temperature *T_C_*: upper operating temperature range of the permanent magnet [°C].

**Table 2:** Average values of the total vector determined on the basis of measurements for the angle of rotation *α* = 27°.

**Measurement Distance *r* [m]**	0.06	0.07	0.08	0.09	0.10	0.11	0.12	0.15
**Total Magnetic Induction *B* [µT]**	781	462	300	206	145	106	75	24

**Table 3:** Estimated values of the *B_x_* component of magnetic induction as a function of rotation angle and measurement distance.

Rotation Angle [°]	Estimated Magnetic Induction Values of Component *B_x_* [µT] as a Function of Rotation Angle and Measurement Distance							
	6.0 cm	7.0 cm	8.0 cm	9.0 cm	10.0 cm	11.0 cm	12.0 cm	15.0 cm
0	45.05	12.53	−7.47	−17.56	−20.37	−18.49	−14.57	−16.58
9	57.68	20.25	−3.03	−15.09	−18.87	−17.31	−13.35	−16.49
18	71.49	29.06	2.32	−11.94	−16.93	−15.88	−12.0	−15.61
27	86.95	37.87	6.79	−9.97	−16.07	−15.19	−10.99	−15.21
36	99.79	44.93	10.21	−8.51	−15.32	−14.36	−9.74	−15.11
45	110.3	51.36	13.86	−6.57	−14.28	−13.64	−9.01	−14.81
54	112.87	53.47	15.64	−5.01	−12.88	−12.38	−7.91	−14.7
63	105.12	50.56	15.38	−4.3	−12.38	−12.73	−9.27	−14.81
72	95.84	45.41	13.03	−4.95	−12.17	−12.28	−8.93	−14.55
81	85.14	40.33	11.4	−4.83	−11.56	−11.99	−9.3	−14.48
90	72.65	34.44	9.56	−4.63	−10.81	−11.6	−9.67	−14.06
99	57.65	27.16	6.94	−5.01	−10.66	−12.02	−11.07	−14.25
108	52.09	23.82	5.14	−5.83	−10.94	−12.08	−11.12	−14.38
117	52.15	23.55	4.72	−6.26	−11.29	−12.28	−11.15	−14.15
126	54.67	24.78	5.13	−6.28	−11.46	−12.43	−11.19	−14.37
135	57.38	25.88	5.31	−6.5	−11.7	−12.47	−10.96	−14.41
144	58.6	26.81	5.98	−6.05	−11.43	−12.33	−10.92	−14.42
153	58.91	26.93	6.05	−5.93	−11.22	−12.03	−10.58	−14.69
162	53.35	23.94	4.66	−6.48	−11.5	−12.39	−11.14	−14.59
171	48.08	20.43	2.45	−7.77	−12.19	−12.71	−11.29	−14.63
180	36.18	13.65	−1.02	−9.38	−13.01	−13.47	−12.33	−14.99
189	23.76	5.75	−5.72	−12.01	−14.44	−14.34	−13.06	−15.44
198	8.13	−3.52	−10.86	−14.78	−16.15	−15.85	−14.76	−15.6
207	−12.58	−15.86	−17.52	−17.94	−17.5	−16.6	−15.62	−16.05
216	−36.55	−30.15	−25.34	−21.9	−19.57	−18.15	−17.38	−16.73
225	−58.51	−43.54	−32.95	−26.0	−21.9	−19.89	−19.2	−17.35
234	−74.63	−54.21	−39.76	−30.23	−24.56	−21.73	−20.66	−17.68
243	−84.68	−61.64	−45.15	−34.08	−27.32	−23.72	−22.15	−18.38
252	−90.1	−66.32	−49.04	−37.19	−29.67	−25.4	−23.27	−18.86
261	−91.65	−67.98	−50.66	−38.65	−30.88	−26.32	−23.9	−18.99
270	−84.58	−64.9	−50.04	−39.25	−31.82	−27.02	−24.11	−19.44
279	−69.15	−57.01	−46.94	−38.78	−32.34	−27.45	−23.94	−19.85
288	−50.01	−46.95	−42.7	−37.72	−32.51	−27.54	−23.29	−19.67
297	−34.17	−37.95	−38.19	−35.83	−31.81	−27.08	−22.57	−19.8
306	−23.29	−31.34	−34.47	−33.87	−30.76	−26.33	−21.79	−19.49
315	−12.35	−24.28	−30.12	−31.28	−29.16	−25.19	−20.78	−19.0
324	−3.97	−18.63	−26.34	−28.68	−27.23	−23.59	−19.33	−18.69
333	5.48	−12.36	−22.38	−26.29	−25.82	−22.71	−18.69	−18.42
342	15.04	−6.15	−18.46	−23.82	−24.16	−21.39	−17.43	−17.71
351	29.47	3.59	−12.08	−19.71	−21.44	−19.43	−15.84	−17.09
360	45.05	12.53	−7.47	−17.56	−20.37	−18.49	−14.57	−16.58

**Table 4:** Estimated values of the *B_y_* component of magnetic induction as a function of rotation angle and measurement distance.

Rotation Angle [°]	Estimated Magnetic Induction Values of Component *B_y_* [µT] as a Function of Rotation Angle and Measurement Distance							
	6.0 cm	7.0 cm	8.0 cm	9.0 cm	10.0 cm	11.0 cm	12.0 cm	15.0 cm
0	−935.49	−549.68	−297.89	−153.37	−89.33	−79.01	−95.63	−39.44
9	−950.14	−557.33	−301.06	−154.06	−89.07	−78.81	−96.01	−39.7
18	−966.92	−566.41	−305.13	−155.28	−89.07	−78.7	−96.39	−39.82
27	−995.92	−581.86	−311.99	−157.49	−89.54	−79.35	−98.08	−39.82
36	−1036.37	−603.45	−321.62	−160.65	−90.32	−80.39	−100.64	−40.18
45	−1089.1	−631.85	−334.48	−164.96	−91.3	−81.49	−103.53	−40.62
54	−1146.77	−663.14	−348.9	−170.09	−92.8	−83.06	−106.96	−41.03
63	−1204.39	−694.65	−363.59	−175.41	−94.3	−84.45	−110.04	−41.36
72	−1261.35	−726.35	−378.87	−181.37	−96.26	−86.0	−113.02	−42.14
81	−1311.41	−755.01	−393.58	−188.08	−99.46	−88.66	−116.62	−42.63
90	−1354.49	−780.13	−406.82	−194.34	−102.44	−90.88	−119.44	−43.43
99	−1375.34	−794.51	−416.45	−200.64	−106.56	−93.71	−121.58	−44.38
108	−1391.35	−805.63	−423.93	−205.54	−109.75	−95.86	−123.16	−45.12
117	−1405.67	−816.13	−431.44	−210.78	−113.34	−98.3	−124.84	−45.82
126	−1418.08	−825.14	−437.81	−215.16	−116.29	−100.27	−126.18	−46.39
135	−1437.46	−837.93	−445.97	−220.29	−119.63	−102.7	−128.23	−46.82
144	−1455.66	−851.1	−455.17	−226.46	−123.58	−105.13	−129.71	−47.57
153	−1475.37	−864.87	−464.47	−232.57	−127.53	−107.73	−131.55	−48.41
162	−1501.96	−882.5	−475.75	−239.6	−131.99	−110.81	−134.0	−48.79
171	−1520.29	−895.22	−484.35	−245.35	−135.86	−113.56	−136.1	−49.32
180	−1519.85	−897.52	−487.81	−248.75	−138.44	−114.91	−136.24	−50.02
189	−1518.18	−898.52	−490.22	−251.61	−141.04	−116.85	−137.36	−50.54
198	−1517.26	−900.76	−493.99	−255.67	−144.5	−119.21	−138.52	−51.16
207	−1503.04	−894.48	−492.56	−256.63	−146.06	−120.22	−138.46	−51.33
216	−1467.98	−876.4	−485.24	−255.12	−146.67	−120.52	−137.3	−51.5
225	−1410.85	−846.73	−472.95	−252.19	−147.13	−120.46	−134.85	−51.23
234	−1340.24	−808.12	−454.95	−245.72	−145.38	−118.9	−131.24	−50.87
243	−1263.45	−765.36	−434.31	−237.65	−142.7	−116.81	−127.3	−50.47
252	−1188.1	−721.53	−411.35	−226.97	−137.78	−113.2	−122.63	−49.03
261	−1111.83	−677.1	−388.02	−216.08	−132.79	−109.64	−118.12	−48.4
270	−1044.69	−638.29	−367.77	−206.58	−128.15	−105.92	−113.33	−47.85
279	−987.19	−603.27	−347.88	−195.87	−122.09	−101.38	−108.61	−46.31
288	−958.3	−583.51	−334.77	−187.34	−116.49	−97.49	−105.61	−45.37
297	−938.54	−568.22	−323.2	−178.81	−110.37	−93.21	−102.67	−44.04
306	−921.0	−554.29	−312.4	−170.67	−104.45	−89.07	−99.88	−42.81
315	−914.17	−547.53	−306.25	−165.49	−100.45	−86.29	−98.19	−42.04
324	−906.27	−540.19	−299.87	−160.35	−96.64	−83.77	−96.74	−40.98
333	−902.66	−536.01	−295.7	−156.6	−93.58	−81.51	−95.26	−40.09
342	−905.73	−535.98	−294.03	−154.41	−91.68	−80.36	−95.0	−40.02
351	−915.33	−540.23	−295.0	−153.75	−90.59	−79.64	−95.02	−40.17
360	−935.49	−549.68	−297.89	−153.37	−89.33	−79.01	−95.63	−39.44

**Table 5:** Estimated values of the *B_z_* component of magnetic induction as a function of rotation angle and measurement distance.

Rotation Angle [°]	Estimated Magnetic Induction Values of Component *B_z_* [µT] as a Function of Rotation Angle and Measurement Distance							
	6.0 cm	7.0 cm	8.0 cm	9.0 cm	10.0 cm	11.0 cm	12.0 cm	15.0 cm
0	784.01	556.34	387.2	267.04	186.3	135.44	104.91	39.78
9	789.37	558.81	387.77	266.52	185.33	134.46	104.17	39.43
18	795.23	562.54	389.94	267.6	185.71	134.45	103.99	39.24
27	800.73	565.87	391.67	268.22	185.62	134.0	103.43	39.18
36	803.88	568.75	394.23	270.44	187.48	135.46	104.49	38.92
45	807.53	573.35	398.71	274.05	189.79	136.36	104.19	39.61
54	812.0	578.98	404.33	278.81	193.18	138.19	104.59	39.74
63	817.59	585.22	410.33	283.94	197.06	140.7	105.89	40.88
72	823.87	592.03	416.78	289.39	201.14	143.3	107.15	41.54
81	831.63	599.9	423.95	295.3	205.48	146.02	108.43	42.17
90	841.41	609.54	432.65	302.53	210.96	149.7	110.55	43.44
99	850.32	618.11	440.41	309.16	216.28	153.67	113.27	44.46
108	859.65	627.24	448.57	315.81	221.14	156.75	114.83	45.73
117	864.45	634.03	455.9	322.6	226.67	160.64	117.07	46.47
126	870.42	639.74	461.14	327.22	230.58	163.82	119.56	47.68
135	869.0	642.89	466.57	333.15	235.77	167.57	121.67	49.07
144	863.3	643.09	470.01	337.78	240.11	170.72	123.33	50.33
153	855.26	641.38	471.97	341.33	243.73	173.45	124.78	51.26
162	843.93	637.93	473.26	344.86	247.64	176.53	126.47	51.78
171	837.23	636.03	474.15	346.97	249.84	178.14	127.25	53.09
180	835.84	636.62	475.98	349.41	252.42	180.49	129.14	53.48
189	833.58	636.15	476.63	350.66	253.84	181.8	130.16	53.84
198	826.68	632.63	475.52	351.12	255.16	183.42	131.64	53.62
207	818.38	627.28	472.35	349.47	254.53	183.39	131.93	54.48
216	808.68	619.79	466.8	345.6	252.04	182.0	131.35	54.46
225	800.04	612.24	460.65	340.99	248.97	180.3	130.71	53.46
234	788.81	602.59	452.81	335.05	244.88	177.88	129.62	53.04
243	779.63	592.68	443.5	327.29	239.21	174.46	128.2	52.2
252	773.39	583.98	434.31	319.06	232.89	170.45	126.41	51.36
261	769.22	576.81	426.1	311.28	226.53	166.01	123.92	49.96
270	765.81	569.64	417.5	303.02	219.8	161.45	121.59	49.06
279	762.45	563.47	410.26	296.0	213.89	157.13	118.91	47.43
288	757.81	557.71	404.24	290.36	209.09	153.39	116.25	46.09
297	756.86	553.74	398.86	284.84	204.29	149.83	114.07	45.15
306	756.97	550.77	394.44	280.21	200.3	146.94	112.34	43.39
315	758.74	550.04	392.04	276.85	196.59	143.38	109.34	43.38
324	762.21	549.11	388.81	272.94	193.13	141.02	108.24	42.2
333	765.86	549.33	387.11	270.48	190.75	139.19	107.1	40.46
342	769.64	549.9	385.72	268.15	188.24	137.07	105.68	40.8
351	774.03	551.33	385.33	266.87	186.77	135.83	104.89	40.21
360	784.01	556.34	387.2	267.04	186.3	135.44	104.91	39.78

**Table 6:** Estimated values of the *B* component of magnetic induction as a function of rotation angle and measurement distance.

Rotation Angle [°]	Estimated Magnetic Induction Values of Component *B* [µT] as a Function of Rotation Angle and Measurement Distance							
	6.0 cm	7.0 cm	8.0 cm	9.0 cm	10.0 cm	11.0 cm	12.0 cm	15.0 cm
0	1221.41	782.19	488.59	308.45	207.61	157.89	142.7	58.42
9	1236.6	789.49	490.93	308.22	206.49	156.81	142.3	58.34
18	1253.97	798.82	495.14	309.62	206.66	156.6	142.3	58.05
27	1280.85	812.54	500.79	311.19	206.72	156.46	142.97	57.9
36	1315.38	830.45	508.88	314.67	208.67	158.17	145.4	57.94
45	1360.29	854.76	520.62	319.94	211.09	159.44	147.16	58.63
54	1409.67	881.95	534.28	326.64	214.7	161.71	149.81	58.98
63	1459.47	909.71	548.46	333.78	218.81	164.59	153.0	60.01
72	1509.62	938.16	563.4	341.56	223.32	167.58	155.99	60.94
81	1555.2	965.16	578.59	350.14	228.58	171.25	159.52	61.69
90	1596.21	990.62	593.96	359.6	234.76	175.51	163.03	63.02
99	1618.0	1007.0	606.17	368.6	241.34	180.39	166.53	64.41
108	1636.33	1021.29	617.22	376.85	247.12	184.14	168.76	65.83
117	1651.03	1033.74	627.7	385.41	253.68	188.73	171.51	66.78
126	1664.8	1044.38	635.88	391.67	258.5	192.47	174.19	68.06
135	1680.7	1056.46	645.44	399.45	264.64	196.93	177.1	69.33
144	1693.42	1067.08	654.31	406.71	270.29	200.87	179.31	70.74
153	1706.36	1077.07	662.22	413.07	275.31	204.54	181.62	72.02
162	1723.65	1089.19	671.07	419.97	280.85	208.8	184.59	72.63
171	1736.24	1098.35	677.81	425.02	284.65	211.64	186.66	73.92
180	1734.9	1100.46	681.55	429.02	288.18	214.39	188.13	74.75
189	1732.14	1100.93	683.76	431.76	290.75	216.59	189.69	75.44
198	1727.87	1100.73	685.76	434.59	293.68	219.33	191.66	75.73
207	1711.44	1092.63	682.67	433.95	293.98	219.91	191.89	76.55
216	1676.39	1073.84	673.8	430.12	292.26	219.04	190.8	76.8
225	1622.96	1045.79	661.03	424.91	290.02	217.75	188.78	76.05
234	1556.93	1009.51	643.12	416.6	285.84	215.06	185.62	75.59
243	1487.04	969.97	622.38	405.9	279.88	211.29	182.02	74.89
252	1420.5	930.61	600.2	393.32	272.21	206.18	177.65	73.46
261	1355.09	892.07	578.53	380.9	264.39	200.68	172.86	72.1
270	1298.07	857.97	558.63	368.83	256.41	194.98	167.96	71.23
279	1249.26	827.46	539.94	357.05	248.4	189.0	162.81	69.2
288	1222.75	808.54	526.59	347.61	241.55	183.82	158.78	67.6
297	1206.17	794.32	514.79	338.22	234.37	178.53	155.12	66.1
306	1192.39	782.03	504.34	329.84	227.98	173.83	151.89	63.99
315	1188.09	776.48	498.39	324.05	222.68	169.23	148.42	63.32
324	1184.19	770.5	491.72	317.85	217.67	165.71	146.45	61.73
333	1183.8	767.6	487.63	313.65	214.03	162.9	144.55	59.86
342	1188.66	767.92	485.35	310.34	210.77	160.32	143.17	59.83
351	1199.1	771.9	485.44	308.62	208.68	158.66	142.41	59.35
360	1221.41	782.19	488.59	308.45	207.61	157.89	142.7	58.42

**Table 7:** Estimated values of the *B_x_* component of magnetic induction as a function of rotation angle and measurement distance.

Rotation Angle [°]	Estimated Magnetic Induction Values of Component *B_x_* [µT] as a Function of Rotation Angle and Measurement Distance							
	6.0 cm	7.0 cm	8.0 cm	9.0 cm	10.0 cm	11.0 cm	12.0 cm	15.0 cm
0	−176.82	−105.17	−58.24	−31.14	−18.99	−16.91	−20.01	−11.52
9	−169.43	−97.32	−50.75	−24.62	−13.8	−13.17	−17.63	−10.36
18	−123.14	−68.92	−34.44	−15.68	−8.64	−9.29	−13.64	−8.69
27	−52.8	−27.37	−12.0	−4.56	−2.92	−4.95	−8.52	−7.21
36	18.48	15.37	11.61	7.52	3.43	−0.33	−3.43	−5.45
45	83.61	54.19	32.89	18.36	9.21	4.1	1.64	−3.39
54	154.43	93.35	51.72	25.87	12.17	6.94	6.55	−2.18
63	205.12	120.14	63.42	29.51	12.96	8.3	10.08	−0.51
72	210.86	123.32	65.08	30.47	13.79	9.36	11.51	0.49
81	182.43	108.92	59.52	29.62	14.61	9.88	10.8	1.31
90	146.53	89.87	51.17	27.1	14.29	9.41	9.1	2.14
99	118.79	73.96	43.09	23.62	12.98	8.61	7.93	2.49
108	105.63	65.26	37.56	20.19	10.81	7.11	6.73	2.26
117	104.51	63.77	35.97	18.71	9.6	6.23	6.21	2.18
126	111.02	66.28	35.95	17.33	7.75	4.53	4.96	1.43
135	120.58	70.15	36.35	16.01	6.01	3.2	4.43	0.91
144	131.79	73.93	35.76	13.47	3.26	1.33	3.88	0.36
153	129.22	70.04	31.43	9.35	−0.21	−1.29	2.07	−1.43
162	115.46	60.01	24.24	4.25	−3.87	−4.01	−0.07	−2.83
171	78.71	36.63	10.09	−4.07	−8.98	−7.78	−3.62	−4.81
180	29.3	6.52	−7.0	−13.2	−14.07	−11.55	−7.6	−6.85
189	−34.37	−31.19	−27.44	−23.39	−19.33	−15.54	−12.3	−8.67
198	−98.84	−67.38	−45.35	−31.03	−22.71	−18.68	−17.22	−11.14
207	−152.8	−98.38	−61.39	−38.52	−26.47	−21.94	−21.61	−12.82
216	−194.91	−122.91	−74.49	−45.13	−30.29	−25.44	−26.06	−15.38
225	−216.35	−135.72	−81.54	−48.74	−32.23	−26.94	−27.78	−16.27
234	−223.42	−140.03	−84.11	−50.39	−33.56	−28.34	−29.42	−17.59
243	−218.85	−137.04	−82.3	−49.42	−33.15	−28.27	−29.56	−18.12
252	−203.27	−127.92	−77.48	−47.14	−32.11	−27.6	−28.81	−18.84
261	−184.98	−117.34	−71.99	−44.62	−30.97	−26.75	−27.69	−18.55
270	−166.55	−106.17	−65.72	−41.37	−29.28	−25.6	−26.51	−18.32
279	−146.53	−94.74	−59.95	−38.9	−28.31	−24.92	−25.47	−18.08
288	−132.42	−86.21	−55.11	−36.22	−26.65	−23.51	−23.9	−17.38
297	−119.15	−78.81	−51.46	−34.65	−25.9	−22.76	−22.74	−16.88
306	−109.43	−73.16	−48.46	−33.15	−25.04	−21.95	−21.72	−16.31
315	−106.25	−71.82	−48.2	−33.35	−25.26	−21.9	−21.26	−15.43
324	−108.49	−73.48	−49.32	−34.0	−25.5	−21.83	−20.97	−15.13
333	−118.31	−79.07	−51.98	−34.81	−25.32	−21.25	−20.38	−14.51
342	−137.1	−88.48	−55.46	−35.11	−24.47	−20.61	−20.56	−13.88
351	−160.69	−99.37	−58.56	−34.28	−22.53	−19.34	−20.73	−12.46
360	−176.82	−105.17	−58.24	−31.14	−18.99	−16.91	−20.01	−11.52

**Table 8:** Estimated values of the *B_y_* component of magnetic induction as a function of rotation angle and measurement distance.

Rotation Angle [°]	Estimated Magnetic Induction Values of Component *B_y_* [µT] as a Function of Rotation Angle and Measurement Distance							
	6.0 cm	7.0 cm	8.0 cm	9.0 cm	10.0 cm	11.0 cm	12.0 cm	15.0 cm
0	84.41	56.99	36.54	21.98	12.19	6.09	2.57	−3.45
9	166.91	102.96	58.79	30.75	15.2	8.5	7.0	−2.76
18	236.97	140.5	75.56	36.13	16.18	9.68	10.59	−2.48
27	264.02	154.7	81.56	37.64	15.97	9.57	11.47	−2.73
36	254.5	147.05	75.51	32.93	12.37	6.88	9.51	−3.25
45	223.27	124.64	59.89	22.34	5.33	2.19	6.25	−5.04
54	161.78	84.49	34.76	7.06	−4.18	−4.5	0.54	−7.66
63	65.53	25.29	0.7	−11.53	−14.71	−12.13	−7.11	−10.39
72	−51.37	−43.34	−36.14	−29.82	−24.43	−20.02	−16.64	−13.16
81	−148.12	−101.06	−67.91	−46.17	−33.38	−27.06	−24.73	−16.84
90	−213.99	−142.03	−92.03	−59.98	−41.87	−33.71	−31.48	−20.38
99	−256.18	−169.66	−109.61	−71.16	−49.5	−39.77	−37.14	−23.42
108	−291.49	−193.71	−125.61	−81.79	−56.86	−45.43	−42.08	−26.57
117	−326.23	−216.57	−140.19	−91.03	−63.07	−50.26	−46.57	−29.85
126	−369.3	−243.49	−156.2	−100.4	−69.07	−55.15	−51.63	−33.07
135	−422.98	−276.21	−174.78	−110.38	−74.69	−59.41	−56.21	−36.04
144	−490.03	−315.87	−196.29	−121.17	−80.44	−63.98	−61.69	−38.83
153	−564.03	−358.45	−218.2	−131.09	−84.93	−67.51	−66.65	−41.37
162	−645.28	−402.98	−239.03	−138.66	−87.07	−69.49	−71.12	−43.46
171	−719.9	−442.21	−255.85	−143.39	−87.39	−70.42	−75.06	−44.3
180	−782.5	−474.03	−268.37	−145.73	−86.31	−70.32	−77.98	−44.86
189	−817.38	−489.73	−272.47	−144.2	−83.51	−68.99	−79.23	−44.5
198	−819.14	−487.28	−268.05	−139.49	−79.68	−66.66	−78.51	−43.8
207	−784.1	−463.63	−252.72	−129.9	−73.73	−62.77	−75.55	−41.93
216	−723.8	−425.92	−230.43	−117.22	−66.18	−57.2	−70.15	−39.54
225	−652.4	−384.0	−207.92	−106.01	−60.16	−52.22	−64.06	−36.97
234	−581.91	−341.73	−184.43	−93.69	−53.21	−46.69	−57.8	−33.88
243	−502.52	−295.75	−160.31	−82.17	−47.3	−41.66	−51.23	−30.87
252	−433.75	−255.24	−138.41	−71.12	−41.2	−36.53	−44.94	−27.19
261	−371.8	−219.11	−119.16	−61.57	−35.94	−31.91	−39.08	−24.04
270	−317.55	−187.43	−102.25	−53.15	−31.29	−27.85	−33.96	−21.27
279	−267.45	−158.61	−87.2	−45.89	−27.34	−24.2	−29.14	−19.03
288	−230.03	−136.37	−75.02	−39.62	−23.83	−21.31	−25.69	−16.85
297	−198.22	−117.76	−64.99	−34.47	−20.78	−18.5	−22.19	−14.83
306	−170.3	−100.66	−55.12	−28.93	−17.35	−15.65	−19.09	−12.86
315	−145.52	−85.15	−45.89	−23.56	−13.98	−12.99	−16.41	−11.39
324	−120.44	−68.92	−35.78	−17.32	−9.88	−9.78	−13.35	−9.3
333	−90.24	−49.37	−23.59	−9.83	−5.02	−6.09	−9.98	−7.81
342	−48.65	−22.25	−6.73	0.26	1.05	−2.01	−6.58	−5.89
351	6.84	12.09	13.2	11.24	7.29	2.44	−2.25	−4.52
360	84.41	56.99	36.54	21.98	12.19	6.09	2.57	−3.45

**Table 9:** Estimated values of the *B_z_* component of magnetic induction as a function of rotation angle and measurement distance.

Rotation Angle [°]	Estimated Magnetic Induction Values of Component *B_z_* [µT] as a Function of Rotation Angle and Measurement Distance							
	6.0 cm	7.0 cm	8.0 cm	9.0 cm	10.0 cm	11.0 cm	12.0 cm	15.0 cm
0	609.32	420.94	284.91	192.0	133.0	98.69	79.84	23.87
9	645.53	437.2	288.99	190.06	129.53	96.54	80.25	22.84
18	694.04	459.74	295.68	188.86	126.29	94.99	81.95	22.5
27	728.95	477.42	302.7	190.42	126.18	95.59	84.28	22.13
36	745.74	486.88	307.36	192.3	126.82	96.07	85.17	22.85
45	748.18	489.52	310.01	194.81	129.08	97.98	86.69	23.22
54	734.36	485.41	311.51	198.73	133.13	100.79	87.76	25.27
63	701.6	474.36	313.24	206.21	141.24	106.32	89.4	26.47
72	670.79	466.53	318.38	216.54	151.2	112.53	90.72	28.29
81	671.1	475.41	331.07	229.41	161.77	119.48	93.89	30.65
90	707.82	503.12	351.74	244.74	173.16	128.05	100.44	33.14
99	770.97	545.32	378.97	261.93	184.22	135.85	106.82	35.87
108	856.87	601.8	414.67	283.96	198.12	145.62	114.92	38.28
117	955.84	665.72	454.2	307.82	213.09	156.53	124.66	42.36
126	1066.81	736.68	497.56	333.65	229.15	168.27	135.21	44.97
135	1182.66	810.37	542.04	359.52	244.64	179.23	145.13	49.07
144	1302.43	888.16	590.37	388.64	262.56	191.72	155.72	52.57
153	1427.79	966.83	637.14	415.51	278.73	203.6	166.9	55.35
162	1548.09	1040.37	679.05	438.03	291.22	212.55	175.92	57.41
171	1655.15	1107.12	718.26	460.09	304.12	221.87	184.86	60.41
180	1732.78	1156.65	748.1	477.17	313.86	228.2	190.19	62.26
189	1783.08	1187.24	765.39	486.35	318.9	231.83	193.95	63.27
198	1804.83	1199.18	771.18	488.87	320.28	233.43	196.35	64.07
207	1791.74	1188.76	763.29	483.29	316.73	231.55	195.72	63.87
216	1738.01	1153.76	741.53	470.27	308.89	226.32	191.49	62.65
225	1657.8	1102.31	710.29	452.19	298.45	219.53	185.88	61.02
234	1559.78	1039.54	672.2	430.12	285.62	211.04	178.72	58.68
243	1445.31	967.76	629.72	406.03	271.54	201.08	169.51	56.52
252	1332.41	896.3	586.87	381.33	256.88	190.73	160.08	53.15
261	1222.7	825.88	543.65	355.46	240.75	178.97	149.57	50.11
270	1121.28	760.12	502.79	330.68	225.22	167.8	139.83	46.75
279	1023.21	697.37	464.38	307.7	210.79	157.11	130.12	43.8
288	936.17	640.98	429.25	286.18	196.96	146.81	120.91	40.75
297	861.17	591.74	398.05	266.69	184.27	137.41	112.69	37.52
306	791.53	546.08	369.2	248.8	172.76	129.01	105.44	34.89
315	729.85	506.24	344.39	233.47	162.69	121.23	98.27	32.36
324	676.2	471.74	323.01	220.33	154.05	114.49	91.98	30.12
333	633.53	443.84	305.42	209.42	146.98	109.25	87.39	28.45
342	604.73	423.99	292.05	200.48	140.84	104.7	83.61	26.32
351	595.75	416.66	286.03	195.49	136.66	101.17	80.63	25.03
360	609.32	420.94	284.91	192.0	133.0	98.69	79.84	23.87

**Table 10:** Estimated values of the *B* component of magnetic induction as a function of rotation angle and measurement distance.

Rotation Angle [°]	Estimated Magnetic Induction Values of Component *B* [µT] as a Function of Rotation Angle and Measurement Distance							
	6.0 cm	7.0 cm	8.0 cm	9.0 cm	10.0 cm	11.0 cm	12.0 cm	15.0 cm
0	640.05	437.61	293.09	195.75	134.9	100.31	82.35	26.73
9	687.95	459.58	299.25	194.09	131.14	97.81	82.46	25.23
18	743.65	485.64	307.12	192.92	127.62	95.93	83.75	24.25
27	777.08	502.6	313.73	194.16	127.22	96.2	85.49	23.44
36	788.19	508.84	316.71	195.24	127.47	96.32	85.77	23.71
45	785.25	508.04	317.45	196.94	129.52	98.09	86.93	24.0
54	767.66	501.47	317.68	200.53	133.75	101.13	88.01	26.5
63	733.9	489.99	319.6	208.63	142.6	107.33	90.25	28.44
72	705.03	484.49	326.97	220.7	153.78	114.68	92.95	31.2
81	711.05	498.09	343.16	235.88	165.82	122.91	97.69	34.99
90	753.84	530.45	367.16	253.44	178.72	132.74	105.65	38.96
99	821.06	575.87	396.85	272.45	191.2	141.81	113.37	42.91
108	911.24	635.57	434.9	296.19	206.4	152.7	122.57	46.66
117	1015.37	702.96	476.7	321.54	222.44	164.52	133.22	51.87
126	1134.37	778.7	522.74	348.86	239.46	177.14	144.82	55.84
135	1261.8	859.02	570.69	376.43	255.86	188.84	155.7	60.89
144	1397.79	945.55	623.17	407.31	274.63	202.12	167.54	65.36
153	1540.59	1033.52	674.2	435.8	291.39	214.51	179.73	69.12
162	1681.16	1117.3	720.3	459.47	303.99	223.66	189.75	72.06
171	1806.64	1192.73	762.53	481.93	316.55	232.91	199.55	75.07
180	1901.5	1250.03	794.82	499.1	325.82	239.07	205.7	77.04
189	1961.8	1284.65	812.91	507.82	330.22	242.38	209.87	77.84
198	1984.48	1296.15	817.69	509.33	330.82	243.48	212.17	78.41
207	1961.76	1279.76	806.37	501.93	326.27	240.91	210.91	77.47
216	1892.77	1235.99	780.08	486.76	317.35	234.82	205.59	75.66
225	1794.64	1175.15	744.58	467.0	306.16	227.26	198.56	73.18
234	1679.72	1103.19	702.1	443.08	292.46	217.99	190.12	70.01
243	1545.75	1021.18	655.0	417.2	277.61	207.29	179.54	66.9
252	1415.9	940.67	607.93	390.76	262.14	196.15	168.74	62.6
261	1291.3	862.48	561.2	363.5	245.38	183.75	157.05	58.59
270	1177.22	790.06	517.27	337.47	229.26	172.01	146.32	54.53
279	1067.69	721.42	476.28	313.53	214.44	160.91	135.75	51.06
288	973.07	660.97	439.23	291.17	200.18	150.2	125.9	47.4
297	891.68	608.47	406.59	271.13	187.24	140.5	117.09	43.73
306	817.01	560.08	376.43	252.66	175.43	131.8	109.34	40.6
315	751.76	518.35	350.76	237.02	165.23	123.87	101.88	37.61
324	695.36	482.38	328.7	223.61	156.46	116.96	95.28	34.96
333	650.78	453.53	310.71	212.52	149.23	111.47	90.28	32.88
342	621.99	433.69	297.34	203.53	142.96	106.73	86.35	30.33
351	617.08	428.51	292.26	198.79	138.69	103.03	83.28	28.33
360	640.05	437.61	293.09	195.75	134.9	100.31	82.35	26.73

## Data Availability

Raw data from this study are available upon request with the agreement of the author.
